# CarboCell G/C offers high and prolonged concentrations of gentamicin and clindamycin in bone tissue following intraosseous injection

**DOI:** 10.5194/jbji-10-327-2025

**Published:** 2025-08-29

**Authors:** Mads Kristian Duborg Mikkelsen, Andrea René Jørgensen, Niranjan G. Kotla, Maiken Stilling, Maria Bech Damsgaard, Christoph Crocoll, Michal Poborsky, Hans Christian Rasmussen, Jonas Rosager Henriksen, Anders Elias Hansen, Mats Bue

**Affiliations:** 1 Aarhus Denmark Microdialysis Research (ADMIRE), Orthopaedic Research Laboratory, Aarhus University Hospital, Aarhus, Denmark; 2 Department of Clinical Medicine, Aarhus University, Aarhus, Denmark; 3 Department of Health Technology, Technical University of Denmark, Kgs. Lyngby, Denmark; 4 Department of Orthopaedic Surgery, Aarhus University Hospital, Aarhus, Denmark; 5 Department of Plant and Environmental Sciences, Faculty of Science, University of Copenhagen, Frederiksberg, Denmark

## Abstract

**Objectives**: The novel local antibiotic-eluting depot technology CarboCell G/C has shown great promise in terms of efficacy in an osteomyelitis pig model. The present study aimed to investigate the initial release kinetics of gentamicin and clindamycin from CarboCell G/C in trabecular bone after a thin-needle intraosseous injection. **Methods**: In the tibial metaphysis of four pigs (both hind legs, n=8 ), microdialysis catheters were placed 5 and 10 mm from an intraosseous injected CarboCell G/C depot of 1.5 mL comprising gentamicin (25 mg) and clindamycin (95 mg). Sampling was performed for 12 consecutive hours to evaluate gentamicin and clindamycin concentrations in trabecular bone and subcutaneous tissue adjacent to the knee joint. Plasma samples were taken to determine systemic spillover. **Results**: Across trabecular bone locations, the mean peak drug concentrations ranged from 471–524 µ g mL^−1^ for gentamicin and from 681–788 µ g mL^−1^ for clindamycin. The mean concentrations at 12 h ranged from 126–321 µ g mL^−1^ for gentamicin and from 255–594 µ g mL^−1^ for clindamycin. Low concentrations of both drugs were found in subcutaneous tissue adjacent to the knee joint ( <1 µ g mL^−1^) and plasma ( <0.2 µ g mL^−1^). **Conclusions**: CarboCell G/C can maintain high local concentrations of gentamicin and clindamycin for at least 12 h after intraosseous injection with negligible systemic spillover. The data bolster the potential benefit of improved local antibiotic delivery systems with flexible administration in target tissues for future therapeutic strategies addressing orthopaedic infections.

## 1 Introduction

Orthopaedic infections present significant clinical challenges due to their multifaceted nature, characterised by significant morbidity, mortality, and treatment resistance, in addition to a profoundly adverse impact on patients' quality of life (Zmistowski et al., 2013; Moore et al., 2015). Current therapeutic strategies include the use of long-term systemic antibiotic therapy coupled with extensive surgical intervention. In recent decades, there has been a growing emphasis on the use of local antibiotics. Local antibiotic application is a logical strategy for treating orthopaedic infections and providing prophylaxis for high-risk patients. Encouragingly, despite being performed in a selected patient population without a control group, local antibiotics have shown substantial efficacy in the management of chronic osteomyelitis by decreasing the recurrence rates of infections (McNally et al., 2022). In the contexts of fracture-related infections (FRIs) and open fractures, local antibiotics yield promising results and have been integrated into current clinical recommendations (Metsemakers et al., 2020; Morgenstern et al., 2018). Yet, data for prosthetic joint infections (PJIs) are more conflicting (Sigmund et al., 2024).

Infectious foci are frequently compromised by inadequate vascularisation, which adversely impacts drug delivery via the systemic route (Bue et al., 2018; Tøttrup et al., 2016). In contrast, local antibiotic delivery technologies can expectedly achieve high drug concentrations at the site of infection. Thus, within infected tissues, local antibiotic delivery may achieve higher drug concentrations than conventional systemic therapy. Ideally, local controlled-release delivery systems should provide sustained release of high antibiotic concentrations, well above relevant minimal inhibitory concentrations (MICs). This property may enable them to eradicate biofilm-associated and bacterial persister populations which require concentrations that are far above normal MIC values and what conventional therapies can achieve. Conversely, concerns exist regarding the potential emergence of bacterial resistance if there is a persistent release of low concentrations of antibiotics from the local application. Nevertheless, a recent study involving 125 patients with recurrent bone and joint infections concluded that treatment with local antibiotics did not correlate with the emergence of resistance (Young et al., 2023).

While local antibiotics hold significant potential, the development of drug-delivery technologies has been limited since the introduction of antibiotic-eluting bone cement in the 1970s (Buchholz et al., 1984). Currently, the range of clinically approved local antibiotic technologies includes modalities such as antibiotic coatings on medical devices and biodegradable antibiotic-eluting biocomposites (Kalbas et al., 2022; Mereddy et al., 2023). Despite their clinical use and approvals, the effectiveness of these technologies in achieving sustained in vivo antibiotic release remains a matter of debate, mainly due to the predominant in vitro nature of the existing knowledge.

CarboCell G/C was developed as a biodegradable polyester system composed of carbohydrate esters, triglycerides, and a solvent. It was specifically designed for local drug delivery, with an emphasis on flexibility in depot distribution by enabling injection through thin needles and placement in and around orthopaedic infections. The therapeutic value of this was demonstrated in an implant-associated osteomyelitis pig model, where CarboCell G/C (comprising gentamicin and clindamycin) eradicated *Staphylococcus aureus* in nine out of nine treated pigs without the use of additional systemic antibiotics. Moreover, histological analyses revealed active bone healing and reorganisation at 3 weeks (Henriksen et al., 2024).

The present study aimed to investigate the initial release kinetics of gentamicin and clindamycin from CarboCell G/C, comprising gentamicin and clindamycin, via microdialysis after a thin-needle 1.5 mL intraosseous injection into the tibial metaphysis of pigs.

## 2 Material and methods

The study was conducted at the Institute of Clinical Medicine, Aarhus University Hospital, Denmark, and animals were acclimatised and kept in pairs for 7 d prior to surgery. Relevant enrichment was provided. The chemical analyses were performed at the DynaMo MS-Analytics Facility, University of Copenhagen, Denmark.

### 2.1 CarboCell G/C

CarboCell is a novel injectable liquid formulation composed of a carbohydrate ester–sucrose octabenzoate, glyceryl, trioctanoate, and ethanol in a 70 : 15 : 15 w/w% ratio. The combination of gentamicin and clindamycin was selected based on in vitro studies suggesting antibacterial synergism against common orthopaedic-device-related infections (Neut et al., 2005; Ensing et al., 2008). The 1.5 mL administered dose of CarboCell G/C contains 25 mg of gentamicin and 95 mg of clindamycin.

### 2.2 Test subjects

Four female pigs (crossbreed of Danish landrace, Danish Duroc, and Danish Yorkshire) with a mean weight of 57 kg (range: 52–61 kg) were included in the study. Before initiating the study, the pigs were premedicated with 1 mL/10 kg Zoletilmix (2.5 mg mL^−1^ tiletamine, 2.5 mg mL^−1^ zolazepam, 2.5 mg mL^−1^ butorphanol, 12.5 mg mL^−1^ ketamine, 12.5 mg mL^−1^ xylazine). Following this, they were intubated and anesthetised with propofol (10 mg mL^−1^ Profast^®^, Baxter Holding B.V.) and fentanyl (50 µ g mL^−1^, B. Braun). The pigs were kept in general anaesthesia for the entire procedure, and HR and MAP were monitored continuously throughout the study and kept above 40 bpm and 65 mmHg.

### 2.3 Surgical procedure

**Figure 1:**
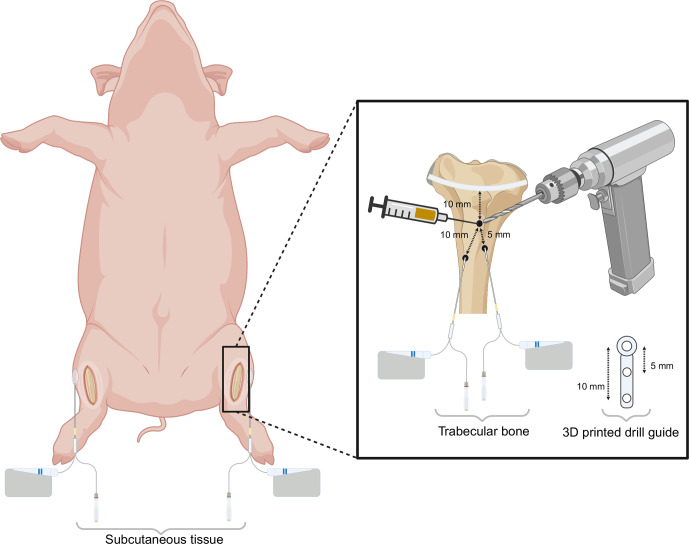
Schematic overview of the bilateral experimental setup. Three holes (Ø 2 mm) were drilled in the medial proximal tibia: one for the injection of CarboCell G/C and two for microdialysis sampling, positioned 5 and 10 mm from the CarboCell G/C injection site. A microdialysis catheter was placed in subcutaneous tissue adjacent to the knee joint. Created in https://biorender.com (last access: 27 June 2025).

In a supine position, a bilateral procedure was performed on the hind legs. A 5 cm surgical incision was made on the medial side of the proximal tibial prominence followed by dissection through the soft tissues down to the bony surface (Fig. 1). The epiphyseal growth plate was identified using fluoroscopy, and approximately 10 mm distal to this point, a Ø 2 mm hole was drilled through the cortical bone to access the trabecular bone. 1.5 mL of CarboCell G/C (containing 25 mg of gentamicin and 95 mg of clindamycin) was then injected into the trabecular bone via a 21G syringe. Two holes, Ø 2 and 25 mm depth, were then drilled 5 and 10 mm parallel to and in a distal direction from the initial hole using a 3D-printed drill guide (Fig. 1). Microdialysis catheters were placed in the drill holes for dynamic sampling of gentamicin and clindamycin released from the CarboCell G/C formulation. Another microdialysis catheter was placed in the subcutaneous tissue adjacent to the knee joint.

A pilot study ( n=2 ) demonstrated that only a negligible systemic spillover of gentamicin and clindamycin ( <0.05 µ g mL^−1^) occurred following an intraosseous injection of CarboCell G/C. Therefore, to reduce the use of experimental animals, a bilateral procedure was performed, which allowed for microdialysis sampling in eight tibias on four animals (both hind legs).

Before the injection of CarboCell G/C, a baseline blood sample was drawn. After placing all microdialysis catheters in both legs, times were synchronised, and sampling commenced. This was performed as close to the application of CarboCell G/C as possible. In this setup, sampling began at a mean of 60 min after the application of CarboCell G/C and continued for 12 h.

At the end of the study, the pigs were euthanised with 100 mg kg^−1^ pentobarbital sodium (Alfasan Nederland BV, the Netherlands) and the hind legs were amputated and CT-scanned to assess the intra-trabecular bone position of the drill holes.

### 2.4 Microdialysis and sampling strategy

Microdialysis catheters (M Dialysis, Sweden) with polyarylethersulphone (PAES) membranes with a cut-off of 20 kDa and lengths of 20 and 30 mm were used for sampling in the trabecular bone and in the subcutaneous tissue, respectively. During the sampling procedure, the catheters were continuously perfused with saline (9 mg mL^−1^, B. Braun) at a flow rate of 1 µ L min^−1^ using a precision pump (CMA 107). For the first 6 h of sampling, samples were collected every 30 min; during the latter 6 h, samples were taken every 60 min. Blood samples were obtained in the middle of the microdialysis sampling intervals, resulting in a total of 18 microdialysates per catheter and 19 blood samples per pig. After the sampling period, the retrodialysis by drug method was employed to individually calibrate the microdialysis catheters. A retrodialysis fluid containing 120 µ g mL^−1^ of clindamycin phosphate was used to calibrate for the absolute concentrations of both gentamicin and clindamycin, with two samples taken at 40 min intervals. The dialysates were promptly stored at -80 °C upon collection. Blood samples were kept at 5 °C for a maximum of 2 h before being centrifuged (4 °C, 3000 RPM, for 10 min). Plasma aliquots were pipetted and subsequently stored at -80 °C.

### 2.5 Chemical analyses

Liquid chromatography coupled to tandem mass spectrometry (LC-MS/MS) was utilised to quantify gentamicin and clindamycin concentrations in microdialysates and plasma, as well as clindamycin phosphate in the calibration microdialysates. For all three drugs, the dilution series was prepared from 0.01 to 10 000 ng mL^−1^; the LOD (limit of detection) was 1 ng mL^−1^ and the LOQ (limit of quantification) 2 ng mL^−1^. For gentamicin, the imprecisions were 7.1 % at 500 ng mL^−1^ and 6.6 % at 5000 ng mL^−1^. For clindamycin, the imprecisions were 8.0 % at 500 ng mL^−1^ and 5.6 % at 5000 ng mL^−1^. Leucine-enkephalin was used as an internal standard for injection control and correction of inter-injection variation. A thorough methodological description of the chemical analyses can be found in the Supplement.

### 2.6 Statistics

Statistical analysis was performed using R (version 4.2.3). Pharmacokinetic parameters across all pigs were calculated using non-compartmental analysis from the *PKNCA* package (version 0.11.0). Data from each leg were treated as an independent observation equalling n=8 . The peak drug concentration ( $C_{max}$ ) and area under the concentration–time curves (AUCs) (employing the trapezoidal rule with linear-up/log-down interpolation) were calculated for all compartments individually. The data are reported as means and 95 % confidence intervals (95 % CI). Normality of data was checked by means of a Q–Q plot.

## 3 Results

All four pigs completed the study, and samples were collected from all 24 catheters. The mean (standard deviation) relative recoveries were 0.57 (0.10) and 0.56 (0.17) for the catheters 5 and 10 mm from CarboCell G/C injection, respectively, and 0.72 (0.14) for the subcutaneous catheters.

**Figure 2:**
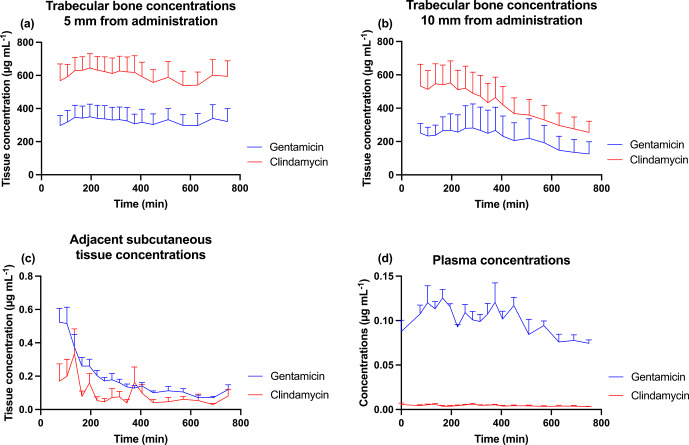
Mean gentamicin and clindamycin concentrations in **(a)** trabecular bone 5 mm from the administration of CarboCell G/C, **(b)** trabecular bone 10 mm from the administration of CarboCell G/C, **(c)** subcutaneous tissue adjacent to the knee joint, and **(d)** plasma. Error bars represent standard error of the mean.

The computed results for $C_{max}$ and AUC are depicted in Table 1, and the resulting concentration–time curves are illustrated in Fig. 2. The subcutaneous tissue adjacent to the knee joint and plasma exhibited low $C_{max}$ and AUC values, over 500-fold lower than those of the two trabecular bone compartments. The pharmacokinetic values were numerically higher at 5 mm from the site of CarboCell G/C administration compared to those at 10 mm, but the mean values remained within each other's 95 % CIs. Across the trabecular bone locations, a mean $C_{max}$ concentration range of 471–524 µ g mL^−1^ was observed for gentamicin and 681–788 µ g mL^−1^ for clindamycin. The mean concentration range at 12 h at 5 and 10 mm from the CarboCell G/C injection was 126–321 µ g mL^−1^ for gentamicin and 255–594 µ g mL^−1^ for clindamycin.

**Table 1:** Mean computed pharmacokinetic parameters for each compartment and drug.

Compartment	Drug	$C_{max}$ [CI 95 %]	AUC_0–last_ [CI 95 %]
Trabecular bone	*Gentamicin*	524 µ g mL^−1^ [333; 714]	3720 µ g h mL^−1^ [2080; 5360]
5 mm from CarboCell	*Clindamycin*	788 µ g mL^−1^ [576; 1000]	6870 µ g h mL^−1^ [4620; 9110]
Trabecular bone	*Gentamicin*	471 µ g mL^−1^ [88; 854]	2580 µ g h mL^−1^ [133; 5030]
10 mm from CarboCell	*Clindamycin*	681 µ g mL^−1^ [344; 1020]	4950 µ g h mL^−1^ [2270; 7630]
Subcutaneous tissue	*Gentamicin*	0.67 µ g mL^−1^ [0.46; 0.87]	2.22 µ g h mL^−1^ [1.70; 2.73]
	*Clindamycin*	0.61 µ g mL^−1^ [0.33; 0.88]	0.98 µ g h mL^−1^ [0.41; 1.54]
Plasma	*Gentamicin*	0.15 µ g mL^−1^ [0.11; 0.19]	1.23 µ g h mL^−1^ [0.94; 1.52]
	*Clindamycin*	0.01 µ g mL^−1^ [0.01; 0.01]	0.10 µ g h mL^−1^ [0.03; 0.07]

All drill holes were verified by computed tomography and were positioned within the trabecular bone of the tibial metaphysis without penetrating the opposite cortex. The exact placement of the CarboCell depot inside the bone could not be determined due to its radiolucent nature.

## 4 Discussion

Intraosseous injection of 1.5 mL CarboCell G/C demonstrates high and prolonged concentrations of gentamicin and clindamycin within porcine trabecular bone throughout the 12 h investigated, with negligible spillover into the systemic circulation. Mapping the antibiotic release kinetics from local applications across various clinical and pathophysiological scenarios is essential for evaluating their therapeutic value and functionality. The pharmacokinetic behaviour of local antibiotic products may vary significantly based on the chosen carrier for delivery, anatomical location, tissue composition, and tissue integrity. Current literature on in vivo pharmacokinetics for other local applications remains limited. This is the first study to directly quantify the initial interstitial tissue concentrations of gentamicin and clindamycin provided by CarboCell G/C injected into the tibial metaphysis of pigs using microdialysis. In contrast, the previous study by Henriksen et al. (2024) reported only the release kinetics of CarboCell G/C based on repeated measurements of the residual depot content; support for the continuous release is provided but without provident insight into the resulting tissue drug exposure or activity over time.

The concentrations of clindamycin were higher than those of gentamicin in trabecular bone. This may be attributed to the higher levels of clindamycin compared to gentamicin in CarboCell G/C (95 mg of clindamycin vs. 25 mg of gentamicin in 1.5 mL). Contrarily, gentamicin concentrations were slightly higher than the concentrations of clindamycin in the adjacent subcutaneous tissue and plasma. The increased systemic concentrations of gentamicin may be explained by the fact that, despite gentamicin being ion-paired with docusate to enhance its lipophilicity and solubility in CarboCell, it remains inherently more hydrophilic than clindamycin, allowing for easier transport into the systemic circulation.

The theoretical discussion surrounding the adequacy of the achieved concentrations for treating infected bone foci presents an intriguing inquiry. Notably, with only a 1.5 mL injection, our results exceed even the most aggressive planktonic MIC targets for the *S. aureus* strains (clindamycin 0.25 µ g mL^−1^ and gentamicin 2 µ g mL^−1^) (EUCAST, 2025) regularly causing orthopaedic infections throughout the 12 h investigated. In fact, the findings surpass pertinent minimum biofilm eradication concentration (MBEC) values for four different clinical *S. aureus* strains (Zaborowska et al., 2017). Our methodological setup (CarboCell G/C injection prior to catheter placement) may have overlooked the true $C_{max}$ , as well as hindering our ability to calculate the time to $C_{max}$ . However, the presented pharmacokinetic profiles of gentamicin and clindamycin appear consistent. Notably, the required duration for maintaining sufficient concentrations is still speculative, and results beyond 12 h were not investigated. However, CarboCell G/C studies in mice and pigs, utilising recovered CarboCell depots for antibiotic quantification, have demonstrated sustained release for a 21 d period (Henriksen et al., 2024). This highlights the important perspective of ascertaining the anticipated kinetic behaviour not only in an in vitro setting, but also in a relevant in vivo environment to elucidate the effect of tissue perfusion and washout kinetics. Prudently, the carrier responsible for delivery dictates the speed of release. In the hydrophobic CarboCell composition, the release is governed primarily by hindered diffusivity, minimising the burst release that is generally challenging for current technologies (Saeed et al., 2019). Injected CarboCell depots have further been demonstrated to be positionally stable (i.e. it remains in the same anatomical position after injection), which is central for securing accurate dosing (Hansen et al., 2020; Henriksen et al., 2024). We present effective containment of high and prolonged concentrations of antibiotics within the designated target tissue (trabecular bone in this study) while maintaining a minimal systemic antibiotic load, which supports the rationale for the optimal application of local antibiotic therapies. Our findings support the positive findings in the treatment of osteomyelitis lesions and the prevention of colonisation on implants in a porcine model, achieved without the use of systemic antibiotics (Henriksen et al., 2024).

A similar microdialysis study investigated trabecular bone concentrations of vancomycin in the metaphysis of a pig tibia following an intraosseous injection of 500 mg diluted in saline (Olsen Kipp et al., 2021). This approximately 5-fold higher dose than that of gentamicin and clindamycin utilised in the present study yielded high concentrations of vancomycin in trabecular bone, although this was unpredictable, less sustained, and with a sharp decline over a 12 h sampling interval, alongside considerable systemic uptake. This underlines the need for a carrier to control the release profile of the antibiotic.

The recently concluded randomised SOLARIO trial, involving 500 patients, is investigating whether a treatment approach incorporating perioperative local antibiotics can reduce the necessity for systemic antibiotics in patients undergoing orthopaedic infection surgeries without implant retention (Dudareva et al., 2019). The results of this study are not yet published but are highly anticipated, as they could significantly alter existing treatment regimens and highlight the potential of local antibiotic applications. This strategy could promote antibiotic stewardship, address the growing concerns regarding antibiotic resistance, and aid in reducing treatment costs.

The precision of the microdialysis catheter placements relative to the injected depots was ensured using a 3D-printed drill guide, resulting in relative uniformity of placement but uncertainty about the exact distance. Nevertheless, our findings suggest that a dose fall-off is observed at longer distances from the depot sites. It is essential to comprehend these gradients within the context of precision in a clinical application setting, recognising that significant variations may arise due to tissue characteristics and inflammation-related factors.

Although describing locoregional flow kinetics is beyond the scope of this paper, future studies are warranted to improve the understanding of this.

One of the knowledge gaps concerning CarboCell G/C is the long-term local tissue toxicology and the rate at which the depot is being resolved. It is uncertain whether a (short- or long-term) local immunological response can influence the bactericidal effect, the local concentrations of the compounds, the healing of a potential fracture, or the osseointegration of implants. Although no signs of immunological reactions have been described in mice and pigs after 3 weeks (Fuglsang-Madsen et al., 2024; Henriksen et al., 2024), long-term studies are warranted, including an accurate estimate of when the depot has been depleted of antibiotics and the depot itself is resorbed.

## 5 Conclusions

In conclusion, CarboCell G/C offers high and prolonged release of gentamicin and clindamycin throughout the 12 h investigated with negligible spillover into the systemic circulation upon injecting only 1.5 mL into trabecular bone. This study contributes valuable in vivo pharmacokinetic data concerning the novel local antibiotic formulation, CarboCell G/C, thereby bolstering the potential benefit of improved local antibiotic delivery systems with flexible administration in bone and soft tissues as an integral component in future therapeutic strategies for orthopaedic infections.

## Supplement

10.5194/jbji-10-327-2025-supplement The supplement related to this article is available online at https://doi.org/10.5194/jbji-10-327-2025-supplement.

## Data Availability

Data are available from the corresponding author upon reasonable request.

## Appendix

The supplement related to this article is available online at https://doi.org/10.5194/jbji-10-327-2025-supplement.
