# Supplementary material for: CarboCell G/C offers high and prolonged concentrations of gentamicin and clindamycin in bone tissue following intraosseous injection
Source: J Bone Jt Infect. 2025 Aug 29;10(4):327–34. doi: 10.5194/jbji-10-327-2025 (PMC12590580; doi:10.5194/jbji-10-327-2025)
Supplement: The supplement related to this article is available online at https://doi.org/10.5194/jbji-10-327-2025-supplement. [file jbji-10-327-2025-supplement.pdf]

Supplement of J. Bone Joint Infect., 10, 327–334, 2025
https://doi.org/10.5194/jbji-10-327-2025-supplement
© Author(s) 2025. CC BY 4.0 License.

JBJI
Journal of Bone and Joint Infection
Open Access

Supplement of

CarboCell G/C offers high and prolonged concentrations of gentamicin and clindamycin in bone tissue following intraosseous injection

Mads Kristian Duborg Mikkelsen et al.

Correspondence to: Mads Kristian Duborg Mikkelsen (makrmi@clin.au.dk)

The copyright of individual parts of the supplement might differ from the article licence.

Liquid chromatography coupled to tandem mass spectrometry (LC-MS/MS) was utilised to quantify gentamicin and clindamycin concentrations in microdialysates and plasma, as well as clindamycin-phosphate in the calibration microdialysates. The analyses were performed on a 1290 Infinity II UHPLC system (Agilent Technologies). Separation was achieved on a Zorbax Eclipse XDB-C18 column (150 x 3.0 mm, 1.8 µm, Agilent Technologies). Formic acid (0.1 %, v/v) in water and acetonitrile (supplied with 0.1 % formic acid, v/v) were employed as mobile phases A and B respectively. The elution profile was: 0.0-1.0 min, 3 % B; 1.0-3.0 min, 3-98 % B; 3.0-4.9 min, 98 % B; 4.9-8.0 min, 98-3 % B and 5.0-6.0 min, 3 % B. The mobile phase flow rate was 400 µL/min. The column temperature was maintained at 40 °C. The liquid chromatography was coupled to an Ultivo Triplequadrupole mass spectrometer (Agilent Technologies) equipped with a Jetstream electrospray ion source (ESI) operated in positive ion mode. The instrument parameters were optimised by infusion experiments with pure standards. The ion spray voltage was set to 3000 V in positive ion mode. The dry gas temperature was set to 325 °C and dry gas flow to 10 L/min. The sheath gas temperature was set to 400 °C and sheath gas flow to 12 L/min. Nebulising gas was set to 40 psi. Nitrogen was used as dry gas, nebulising gas and collision gas. Multiple reaction monitoring (MRM) was used to monitor precursor ion → fragment ion transitions. MRM transitions and other parameters such as fragmentor voltage and collision energies were optimised using reference standards. Both Q1 and Q3 quadrupoles were maintained at unit resolution. Mass Hunter Quantitation Analysis for QQQ software (Version 10, Agilent Technologies) was used for data processing. Linearity in ionisation efficiency was verified by analysing dilution series that were also used for quantification. For all three drugs, the dilution series was prepared from 0.01 ng/ml to 10000 ng/ml and LOD was 1 ng/ml and LOQ 2 ng/ml. For gentamicin, the imprecisions were 7.1% at 500 ng/mL and 6.6% at 5000 ng/mL. For clindamycin, the imprecisions were 8.0% at 500 ng/mL and 5.6% at 5000 ng/mL. Leucine-Enkephalin was used as an internal standard for injection control and correction of inter-injection variation.

Microdialysates were prepared with milliQ grade water containing 0.1 % formic acid (v/v) and 1000 ng/ml leucine-enkephalin as internal standard (5 µl microdialysate were added to 95 µl water with formic acid and IS). Samples were further diluted with deionized water containing 0.1 % formic acid (v/v) to final dilutions of 200-fold and 2000-fold prior to analysis.

Plasma samples were cleaned by using 30 kDa cutoff filters (AcroPrep Advance 96-well filter places for Ultrafiltration - 350 µL, Omega 30K MWCO, Cytiva) according to the manufacturer's manual. Prior to application to the filters, the samples were centrifuged at 21000 x *g* for 10 min at 10 °C. 200 µl of the supernatant was applied to the filter plates. Filter plates were centrifuged for 60 min at 1500 x *g*. An aliquot of the flow through was mixed with milliQ grade water containing 0.1 % formic acid (v/v) and 1000 ng/ml leucine-enkephalin as internal standard (10 µl sample was added to 190 µl). Diluted samples were directly subjected to analysis by LC-MS/MS.
